# *Clostridium sticklandii*, a specialist in amino acid degradation:revisiting its metabolism through its genome sequence

**DOI:** 10.1186/1471-2164-11-555

**Published:** 2010-10-11

**Authors:** Nuria Fonknechten, Sébastien Chaussonnerie, Sabine Tricot, Aurélie Lajus, Jan R Andreesen, Nadia Perchat, Eric Pelletier, Michel Gouyvenoux, Valérie Barbe, Marcel Salanoubat, Denis Le Paslier, Jean Weissenbach, Georges N Cohen, Annett Kreimeyer

**Affiliations:** 1 CEA, DSV, Institut de Génomique, Genoscope, 2 rue Gaston Crémieux, F-91057 Evry, France; 2 CNRS-UMR 8030 F-91057 Evry, France; 3 UEVE, Université d'Evry, F-91057 Evry, France; 4 Institut Pasteur, 28 rue du Dr. Roux, F-75724 Paris cedex 15, France; 5 Institute of Biology/Microbiology, University of Halle, Kurt-Mothes-Str. 3, D-06120 Halle, Germany

## Abstract

**Background:**

*Clostridium sticklandii *belongs to a cluster of non-pathogenic proteolytic clostridia which utilize amino acids as carbon and energy sources. Isolated by T.C. Stadtman in 1954, it has been generally regarded as a "gold mine" for novel biochemical reactions and is used as a model organism for studying metabolic aspects such as the Stickland reaction, coenzyme-B12- and selenium-dependent reactions of amino acids. With the goal of revisiting its carbon, nitrogen, and energy metabolism, and comparing studies with other clostridia, its genome has been sequenced and analyzed.

**Results:**

*C. sticklandii *is one of the best biochemically studied proteolytic clostridial species. Useful additional information has been obtained from the sequencing and annotation of its genome, which is presented in this paper. Besides, experimental procedures reveal that *C. sticklandii *degrades amino acids in a preferential and sequential way. The organism prefers threonine, arginine, serine, cysteine, proline, and glycine, whereas glutamate, aspartate and alanine are excreted. Energy conservation is primarily obtained by substrate-level phosphorylation in fermentative pathways. The reactions catalyzed by different ferredoxin oxidoreductases and the exergonic NADH-dependent reduction of crotonyl-CoA point to a possible chemiosmotic energy conservation *via *the Rnf complex. *C. sticklandii *possesses both the F-type and V-type ATPases. The discovery of an as yet unrecognized selenoprotein in the D-proline reductase operon suggests a more detailed mechanism for NADH-dependent D-proline reduction. A rather unusual metabolic feature is the presence of genes for all the enzymes involved in two different CO_2_-fixation pathways: *C. sticklandii *harbours both the glycine synthase/glycine reductase and the Wood-Ljungdahl pathways. This unusual pathway combination has retrospectively been observed in only four other sequenced microorganisms.

**Conclusions:**

Analysis of the *C. sticklandii *genome and additional experimental procedures have improved our understanding of anaerobic amino acid degradation. Several specific metabolic features have been detected, some of which are very unusual for anaerobic fermenting bacteria. Comparative genomics has provided the opportunity to study the lifestyle of pathogenic and non-pathogenic clostridial species as well as to elucidate the difference in metabolic features between clostridia and other anaerobes.

## Background

Amino acids are used as important carbon and energy sources for some microorganisms. This strategy represents an advantage in protein-rich environments, but it is used even when crucial metabolic processes (e.g. protein biosynthesis), may be impaired. A variety of anaerobic bacteria have developed specific pathways to degrade amino acids by fermentation processes. Anaerobic amino acid utilization was extensively studied in the seventies, especially in Clostridia [1,2]. The genus *Clostridium *consists of a large group of Gram-positive, anaerobic bacteria which belong to the Firmicute phylum [3]. This genus employs pathways and enzymes with unique activities mostly involved in amino acid degradation, such as B12-dependent aminomutases, selenium containing oxidoreductases and extremely oxygen-sensitive 2-hydroxyacyl-CoA dehydratases [4-6]. A striking metabolic feature of Clostridia is the fermentation of amino acids *via *the Stickland reaction, described in 1934 by L. H. Stickland [7]. It is characterized by the oxidation of one amino acid coupled to the reduction of another. In this process energy is mainly conserved by ATP formation *via *substrate-level phosphorylation (SLP). However, little is known about other systems by which the Stickland reaction contributes to energy and growth, despite many studies of this aspect of clostridial metabolism [6,8-11]. In this context, one of the best biochemically studied clostridial species is *Clostridium sticklandii*. After its isolation from San Francisco Bay black mud in 1954 [12,13], it was described to be a specialist in amino acid degradation [14,15]. More specifically, *C. sticklandii *can utilize threonine, arginine, lysine and serine as reductants in the Stickland reaction, whereas glycine and proline are used as oxidants [1,2,16]. It has been described that ornithine/proline or ornithine/lysine can be used as amino acid pairs in the Stickland reaction. Aromatic and branched-chain amino acids also seem to be degraded, but the pathways involved are still unknown [17,18]. Two amino acids, glutamate and alanine, are not utilized. Stadtman has stated that formate, when added to the amino acid fermentations, increased growth yield [12,16]. Besides the degradation of amino acids, it has also been reported that *C. sticklandii *catabolises purines [19,20] and slightly ferments carbohydrates such as glucose, maltose and galactose [21]. However, these compounds are not or only minor substrates for energy and growth.

To get a general view of amino acid catabolising microorganisms, we performed a preliminary bioinformatic analysis (Blast searches) of complete sequenced bacterial genomes in which specific enzymes involved in amino acid degradation pathways are present. The results showed that *Clostridium *spp. possess most of these pathways (data not shown). Since the biochemistry of amino acid fermentation has been principally studied in *C. sticklandii*, this bacterium is an appropriate candidate for learning more about the fascinating process of anaerobic amino acid degradation. We have sequenced and analyzed its genome. Together with supplemental experimental procedures these studies have confirmed and extended our knowledge of amino acid degradation. They have also revealed previously unknown metabolic capacities of *C. sticklandii *that contribute to an improved understanding of its energy-conserving system, particularly the processes of chemiosmotic ATP generation. Moreover, a hitherto unrecognized selenoprotein and an unusual combination of two distinct CO_2_-fixation pathways have been discovered. Finally, this work has provided the opportunity for a comparative study of other clostridial genomes, notably the most closely related pathogenic strain, *Clostridium difficile *[6,21,22]. Together, these results help to elucidate the biochemistry, energetics and growth of clostridia and other anaerobic amino acid degrading bacteria.

## Results and discussion

### Global genomic and metabolic features

The major features of the *C. sticklandii *genome are listed in Table 1 (comparison with other clostridial species - Additional file 1). Its genome consists of a single circular chromosome of 2,715,461 bp, which carries 2573 coding sequences (CDS). The number of CDS in such a small genome and the low level of repeated regions (2.1%) explain the high protein coding density of 89.2%. Of the 2573 CDS, 33% were annotated as conserved hypothetical or hypothetical proteins. Two members of the genus *Alkaliphilus *(*A. metalliredigens *QIMF and *A. oremlandii *OhILas) and *Clostridium difficile *630 share the highest number of orthologous genes with *C. sticklandii *(Additional file 2).

**Table 1:** Genomic features of the *C. sticklandii *DSM 519 genome.

Features	
Complete genome size, Mb	2.7
G+C content (%)	33.3
Repeat regions (%)	2.1
Number of CDS	2573
Protein coding density (%)	89.2
Average CDS length (bp)	945
Number of tRNAs	59
Number of rRNA clusters	6

Annotation of the *C. sticklandii *genome reveals that most biosynthetic pathways considered as essential for viability seem to be present (e.g., biosynthesis of amino acids, purines, pyrimidines and cofactors). However, it should be noted that genes involved in synthesis of biotin and pantothenate are absent, confirming the absolute requirement of these two vitamins for growth observed by Golovchenko et al. [23]. These authors also report a requirement for cyanocobalamin and thiamin. The absence of some genes involved in the biosynthesis of these cofactors is in agreement with these results. Pyridoxal phosphate synthesis proceeds through the proteins encoded by *pdxS *and *pdxT *[24] and not by the pathway existing in *E. coli *and many other bacteria. *C. sticklandii *is unable to assimilate sulphate, since genes involved in this pathway are absent (*cysN*, *cysD*, *cysC*, and *cysH *). This inability is also found in some other clostridia (Additional file 1). In *C. sticklandii*, the sulphur requirement for cysteine biosynthesis can be met by sulphide (data not shown) since genes coding for serine O-acetyltransferase (*cysE*) and O-acetylsulfhydrylase (*cysK*) are present. The only genes of the upper portion of the tricarboxylic acid cycle found with certainty in this bacterium are the adjacent genes encoding *Si*-citrate synthase and isocitrate dehydrogenase. Since glutamate is excreted (see section below), there is no need for glutamate formation *via *the tricarboxylic acid cycle. Glutamate could be formed by histidine degradation since all the genes coding for the enzymes of histidine biosynthesis and degradation are present. Additionally, 2-ketoglutarate could be formed by reductive carboxylation of succinyl-CoA using reduced ferredoxin by one of the many 2-ketoacid ferredoxin oxidoreductases. All genes involved in the glycolysis pathway were detected. However, we found that *C. sticklandii *is not able to grow on glucose (data not shown) although a slight fermentation of glucose has been described [21]. This is explained by a defect in some transport components [25]. Indeed, genome analysis shows the absence of genes coding for protein II of the PTS system. The genes of the non-oxidative pentose phosphate pathway are present, accounting for the synthesis of hexose and pentose phosphates respectively. A cluster of genes encoding a ribose transporter and a ribokinase could lead to ribose 5-phosphate, which can be completely converted into glycolytic intermediates.

As a proteolytic species, *C. sticklandii *contains about 12 proteases and 38 peptidases and is highly dependent on the utilization of amino acids (see section below). Annotation of its genome has confirmed the presence of several biochemically described amino acid degradation pathways (Additional file 3). Similar amino acid degradation pathways are found in the metal-reducing strain *Alkaliphilus metalliredigens *QIMF, in the arsenic-resistant strain *Alkaliphilus oremlandii *OhILas [26,27] as well as in the pathogenic strains of *C. difficile *and *C. botulinum *(Additional file 4). It should be noticed that arginine deiminase is missing in the genome of *C. difficile*, although this species contains all the genes involved in the subsequent steps in the arginine breakdown pathway. The genes coding for several transporters for amino acids were found in *C. sticklandii *(e.g., ABC transporters for oligopeptides, methionine, and branched-chain amino acids, a serine/threonine symporter as well as an arginine/ornithine antiporter). The degradation of the purines (adenine, xanthine and uric acid) by *C. sticklandii *has been reported [19,20]. However, xanthine dehydrogenase, the key enzyme of purine degradation, was not found in this genome. We cannot exclude that purine compounds are converted by a totally different 2-ketopurine dehydrogenase. This protein has been purified from several species of peptostreptococci [28], but the gene is not known.

### Preferential and sequential amino acid utilization

**Figure 1:**
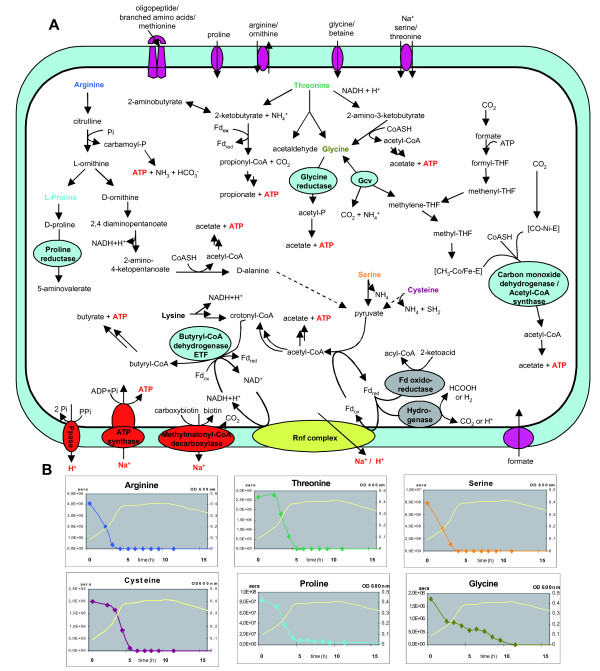
**Presentation of relevant metabolic features and amino acid utilization**. (A) Genome-based model of amino acid metabolism and energy conservation processes of *C. sticklandii*. (B) LC-MS analyses of amino acid utilization. The presence of each amino acid in the medium was checked at different phases of growth (colour graphs). The growth kinetic is represented by a yellow graph. Only the curves for rapidly used amino acids are shown.

Important features of amino acid metabolism by *C. sticklandii *are depicted in Figure 1A. It oxidizes ornithine, arginine, threonine, serine and reduces proline and glycine [2,12,15]. *C. sticklandii *can utilize glycine and ornithine both as an electron donor and an electron acceptor in the Stickland reaction. Ornithine can be reduced to 5-aminovaleric acid through the formation of D-proline as an intermediate, or it can be oxidized to acetate, D-alanine and ammonia [15,29,30]. Glycine can be directly oxidized by NAD^+ ^into methylene-THF, CO_2 _and ammonia *via *the glycine cleavage system (gcv-system) or reduced to acetyl phosphate by glycine reductase [31]. Electrons from the gcv-system can be directly utilized by glycine reductase as observed for *Eubacterium acidaminophilum *[32].

Since a large variety of amino acids can be utilized by *C. sticklandii *as nutrient and energy sources, it was interesting to determine which amino acids are preferentially metabolized by this bacterium. For this purpose *C. sticklandii *was cultivated in a defined medium with the amino acids at the same concentration (see Methods). Using Liquid Chromatography-Mass Spectrometry (LC-MS) analysis (Figure 1B and Additional file 5) we found that arginine, serine, threonine, cysteine, proline and glycine disappear rapidly from the medium in the exponential growth phase. In contrast, lysine, histidine, asparagine and valine disappear at the onset of the stationary growth phase. The concentrations of the aromatic amino acids remained stable: they were not metabolized under our growth conditions. Glutamate, aspartate and L- and D-alanine are excreted. These results show that *C. sticklandii *seems to prefer certain amino acids and degrades them in a specific order.

Subsequently, we tried to associate amino acid utilization with the analysis of genes coding for proteins involved in the degradation pathways. The rapid utilization of threonine can be explained by the presence of three pathways through which this amino acid is catabolized. Threonine dehydrogenase oxidizes threonine into 2-amino-3-ketobutyrate [33] which is split into glycine and acetyl-CoA. Threonine aldolase converts threonine into glycine and acetaldehyde, whereas threonine dehydratase transforms threonine into ammonia and 2-ketobutyrate. The latter compound is oxidized to propionate and CO_2 _or reductively aminated to 2-aminobutyrate (Figure 1A), typically formed by *C. sticklandii *[1] and detected in extracellular medium by LC-MS (data not shown). Arginine is quickly metabolized through the arginine deiminase pathway into ornithine *via *citrulline. This is a generally favoured pathway by anaerobes, for they can conserve energy without any redox reaction by splitting the deaminated intermediate citrulline by ornithine carbamoyl-phosphate transferase and conserve ATP by carbamate kinase. As expected, *C. sticklandii *contains all the genes involved in the arginine deiminase pathway as well as the ornithine oxidative and reductive pathways [29,30]. Like *C. botulinum *and *C. sporogenes *[34], *C. sticklandii *possesses an ornithine cyclodeaminase that directly produces proline from ornithine. Dehydration of serine leads to pyruvate which is decarboxylated into acetyl-CoA by pyruvate ferredoxin oxidoreductase. The rapid utilization of cysteine may be correlated with its degradation to pyruvate, ammonia and hydrogen sulphide by a putative L-cysteine sulphide lyase. In the degradation of lysine into acetate, butyrate, and ammonia [35-37], the highly exergonic reduction of crotonyl-CoA to butyryl-CoA contributes to energy conservation [38]. However, lysine is not used in the exponential growth phase (Additional file 5). One could hypothesize that during this growth phase crotonyl-CoA is formed from the condensation of two acetyl-CoAs *via *butyrate fermentation, since all the genes involved in this pathway are present in *C. sticklandii*. Acetyl-CoA could be generated from serine, arginine and cysteine during the exponential growth phase. Thus, after depletion of these amino acids (at the beginning of the stationary growth phase) crotonyl-CoA is produced from lysine fermentation. It has been reported that *C. sticklandii *degrades aromatic and branched chain amino acids by oxidative means [14,17,18]. However, under our experimental conditions, aromatic amino acids do not appear to be metabolized (Additional file 5) whereas valine, leucine, and (or) isoleucine (the two latter compounds are not distinguishable by mass spectrometry) disappear from the medium. They are probably transaminated to the corresponding 2-ketoacids, which are further degraded by the branched-chain fatty acid ferredoxin oxidoreductase. Alanine, which was not added to the medium, may be formed by the breakdown of ornithine. This amino acid is excreted in the exponential phase, but disappears slowly in the stationary growth phase. Stadtman has shown that the Stickland reaction amino acid pair, alanine and glycine, is not operative in *C. sticklandii *[16]. A gene coding for alanine dehydrogenase is actually absent in *C. sticklandii*. Glutamate might be formed by degradation of histidine but in contrast to other clostridial species, especially *C. tetanomorphum *and *C. cochlearium *[39], glutamate is not used by *C. sticklandii *in accordance with previous results [1,2]. Genes encoding methylaspartate mutase or 2-hydroxyglutaryl-CoA dehydratase, the key enzymes of the two major glutamate fermentation pathways [40], have not been detected in the genome.

Based on the results of amino acid utilization, we tried to grow *C. sticklandii *in three different media. The following amino acid combinations were utilized: the first contained amino acids utilized in the exponential growth phase, the second contained those utilized in the exponential and stationary growth phase and the third was equivalent to the second complemented with amino acids that apparently were not metabolized (Additional file 6). Surprisingly, no growth was observed in the medium with the second amino acid combination. Similar experiments have been performed by Golovchenko *et al. *using *C. sticklandii *strain CSG [23]. Thus, it appears that the combination of amino acid influences the growth of *C. sticklandii*. There are probably regulatory properties implying that the presence or absence of some amino acids could repress the utilization or the biosynthesis of other amino acids. In particular, it has been shown that in some clostridial species that perform the Stickland reaction, the type of amino acid pair is quite important for growth [41].

### Discovery of PrdC as a selenocysteine-containing protein

**Figure 2:**
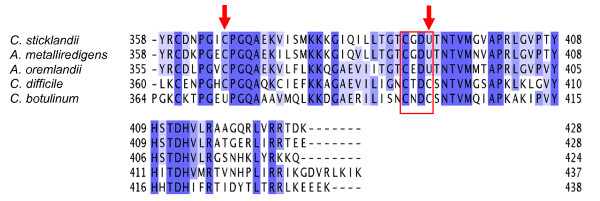
**Multiple sequence alignments of the C-terminal sequence of PrdC of several Clostridiales**. Conserved residues are highlighted. The red arrows show the position of the selenocysteine (U). The redox centre is boxed in red.

Selenoproteins are characterized by the replacement of a cysteine residue by a selenocysteine (Sec) encoded by the UGA stop-codon. A complex machinery is required for translational insertion of Sec in response to the UGA codon. In fact, the first selenoprotein, glycine reductase A (GrdA), was discovered in *C. sticklandii *[42,43]. Analysis of the *C. sticklandii *genome revealed that all known components of the Sec insertion machinery (Additional file 3) and the Sec tRNA are present. Using the gene fusion-fission functionality of MaGe [44], we identified eight selenoproteins in *C. sticklandii *(Table 2). Interestingly, one of these proteins, PrdC, had never been described as a selenoprotein before, although the proline reductase genes have been studied in detail in both *C. difficile *and *C. sticklandii *[22,45,46]. PrdC is located in the D-proline reductase operon and is annotated as an electron transfer protein [45]. This role has also been suggested by Eversmann [47]. Evidence for the exact biochemical role of PrdC is now provided by the discovery of a C-terminal redox center -CxxU-. The amino acid sequence of PrdC exhibits homology to RnfC, the protein involved in the NADH-dependent Rnf electron transfer complex. Recently, Kim *et al. *have identified RnfC as a selenoprotein in *Alkaliphilus oremlandii *OhILAs (accession no. YP_001511593). However, the genomic context and the alignment of this sequence with the PrdC of several clostridial species (Figure 2) show that this selenoprotein (RnfC) is actually PrdC. This alignment also shows that the selenocysteine-residue is present in all PrdC proteins in the terminal -CxxU-motif with two exceptions: there is a deviation of the selenocysteine position in PrdC of *C. botulinum*, and PrdC of *C. difficile *does not contain a selenocysteine but a cysteine residue, thus emphasizing that the presence of selenocysteine in PrdC is not a necessity, but is probably catalytically advantageous as has been shown e.g. for methionine sulphoxide reductases [48].

**Table 2:** Selenocysteine containing proteins in *C. sticklandii*.

Proteins	CDS number	Sec position (protein length)
**Known selenoproteins:**		
Formate dehydrogenase (FdhA)	CLOST_0829	134 (699)
Betaine reductase (GrdH)	CLOST_1057	352 (440)
Glycine reductase (GrdA)	CLOST_1112	44 (158)
Glycine reductase (GrdB)	CLOST_1113	350 (436)
Proline reductase (PrdB)	CLOST_2232	152 (242)
Peroxiredoxin	CLOST_2406	84 (240)
Putative glutathione peroxidase	CLOST_2446	35 (161)
**New selenoprotein:**		
Electron transfer protein (PrdC)	CLOST_2236	392 (428)

### New insights into the D-proline reductase mechanism

**Figure 3:**
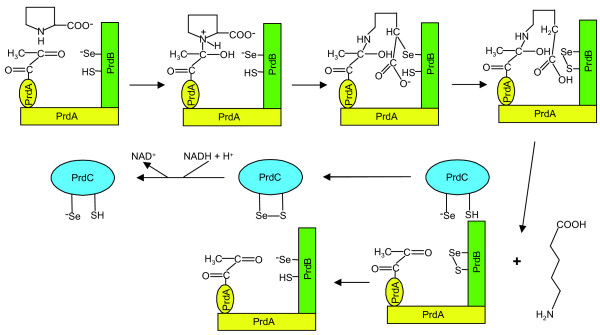
**Proposed mechanism of D-proline reductase (adapted from Kabisch **[45]**)**. PrdA is shown after the hydrolytic processing of the proprotein to form a pyruvoyl group that binds D-proline [46].

D-Proline acts as the electron acceptor in the Stickland reaction. The D-proline reductase has been biochemically studied only in *C. sticklandii *[46,49] and in *C. difficile *[22]. It catalyzes the reductive ring cleavage of D-proline into 5-aminovalerate, which is subsequently excreted. In *C. sporogenes*, the reduction of D-proline was described to be coupled to transmembrane proton ejection. However, it has not been demonstrated whether this proton gradient is coupled to ATP synthesis [50]. This is possibly due to a very low proton ejection. The D-proline reductase activity of *C. sticklandii *was detected in the cytoplasmic fraction [46]. In contrast to ATP formation by the glycine reductase reaction [8], no obvious energy conservation step was identified in the D-proline reductase reaction [45,46,51]. Surprisingly, despite this energetic disadvantage, *C. sticklandii *preferentially uses proline when glycine and proline are both present in the synthetic medium [31,52]. It seems likely that D-proline reductase is used as an electron sink and plays a role in the redox-balance of the cell [51]. NADH has been suggested as the natural electron donor [51,53]. Genes involved in D-proline reduction are clustered together in an operon and the organization of the genes is conserved in all the organisms studied (Additional file 7). PrdF encodes the proline racemase necessary for D-proline generation in order to discriminate it from the L-proline used for protein synthesis. PrdA undergoes a post-translational modification forming a catalytically active pyruvoyl group [47,54] that binds the D-proline [45,46] (Figure 3). The dissociated, nucleophilic selenocysteine moiety in PrdB could then attack the α-C-atom of proline. This may lead to a reductive cleavage of the C-N-bond of the pyrrolidine ring and the formation of a selenoether. The selenoether would then be cleaved by a cysteine moiety of PrdB resulting in a mixed selenide-sulphide group. PrdC contains NADH- and FMN-binding sites as well as an iron-sulphur cluster as suggested before [46,51,53] and has been hypothesized to function as an electron transport protein [45,47]. With the detection of the potential redox-active center including a selenocysteine in PrdC, we now propose the following extension of the reaction: PrdC will use NADH to reduce its special terminal sulphide-selenide group into the thiol-selenol form (Figure 3). The latter seems to be optimal to reduce the oxidized selenide-sulphide bond of PrdB by an exchange reaction. A similar type of dithiol-disulphide exchange reaction (partly with an involvement of selenocysteine) occurs during glycine reduction by NADPH *via *thioredoxin reductase, thioredoxin, GrdA and GrdB, where selenocysteine replaces one cysteine in GrdA and GrdB [8,55]. PrdD and PrdE correspond quite significantly in their sequences to the middle and C-terminal part of proprotein PrdA. Both genes are present in an operon-like structure in all genomes of organisms containing a D-proline reductase (Additional file 7). However, both genes are absent in the second set of D-proline reductase genes in *C. botulinum*, pointing to a more indirect, protein-stabilizing role as shown for the related two proteins derived from proprotein GrdE in glycine reductase [8,55]. Finally, 5-aminovalerate is formed by hydrolysis to regenerate the pyruvoyl moiety [46]. PrdG is a potential membrane-bound protein and might be responsible for the excretion of 5-aminovalerate.

### Oxidative stress response

Clostridia are described in textbooks as obligate anaerobes since oxygen is harmful or lethal to these bacteria. For example, pyruvate ferredoxin oxidoreductase, a central enzyme of their metabolism is quickly inactivated in the presence of oxygen. Apparently, this results from damage to its exposed iron-sulphur sites [56]. However, some obligate anaerobes can tolerate small amounts of oxygen thanks to the presence of some proteins involved in oxygen detoxification [10]. In *C. acetobutylicum *genes coding for some of these proteins are under control of the peroxide repressor PerR, whose deletion results in a prolonged aerotolerance [57]. Genome analysis of *C. sticklandii *has revealed the presence of a protein similar to PerR as well as several proteins involved in the oxidative stress response. Beside the classical Mn-superoxide dismutase and superoxide reductase, *C. sticklandii *possesses genes encoding an alkyl hydroperoxide reductase, a regular rubrerythrin, two putative glutathione peroxidases, a seleno-peroxiredoxin as well as a thioredoxin-dependent peroxidase. The methionine sulfoxide reductases A and B involved in the repair of oxidative damaged methionine have also been detected. This well-supplied protection system could enhance the survival of *C. sticklandii *under microaerophilic conditions. In fact, we observed slight growth when *C. sticklandii *was cultivated in an aerobic atmosphere (data not shown).

### Energy conservation

The energy conservation of *C. sticklandii via *amino acid fermentation and especially by the Stickland reaction, has been the subject of many investigations. In this context, glycine reductase seems to play a central role [8]. The reduction of glycine leads to ATP formation by SLP with acetate as the final product. SLP is also an important step in some fermentation processes (Figure 1A). Energy conservation *via *electron-transport phosphorylation (ETP) in *C. sticklandii *is performed by the Rnf complex. This complex, that was recently discovered in various bacteria and archaea, is a membrane-bound system showing strong similarity to the *Rhodobacter capsulatus *nitrogen fixation and some NADH:quinone oxidoreductase systems. It catalyzes the electron flow from reduced ferredoxin to NAD^+^. This process is coupled to ion-translocation across the membrane, thus allowing chemiosmotic energy generation. Proton-pumping Rnf clusters have been integrated into the energy-conserving systems of *Clostridium tetani *[58], *Clostridium kluyveri *[38] and *Acetobacterium woodii *[59]. In *C. sticklandii*, reduced ferredoxin is formed in several metabolic processes such as the highly exergonic NADH-dependent reduction of crotonyl-CoA to butyryl-CoA in the butyrate and lysine fermentation pathways [60,61] and the reactions catalysed by numerous ferredoxin oxidoreductases present in the *C. sticklandii *genome (Figure 1A). In addition to the possible ion-gradient driven chemiosmotic ATP generation *via *the Rnf complex, *C. sticklandii *possesses a Na^+^-dependent F0F1-ATPase and an additional V-ATPase. This is a quite unusual combination for bacteria [62]. Further energy conservation can be provided by a reaction catalyzed by a putative methylmalonyl-CoA decarboxylase. This membrane-bound protein couples the decarboxylation of methylmalonyl-CoA to the efflux of Na^+ ^ions, which re-enter the cell by the ATPase systems. A third chemiosmotic energy-conserving process is catalyzed by a membrane-bound pyrophosphatase [63]. An alternative energy-conserving system in anaerobes is provided by the presence of hydrogenases. The *C. sticklandii *genome contains two cytoplasmic iron-only hydrogenases (HydA and HymABC). The multisubunit hydrogenase HymABC exhibits similarity with the hydrogenase identified in *Eubacterium acidaminophilum *[64]. The structural gene for the second hydrogenase HydA is clustered together with genes coding for a formate dehydrogenase (FdhA, FdhB), making it possible for these proteins to act as a formate hydrogen lyase. Formate addition to the culture medium increased growth efficiency of *C. sticklandii *[12]. A formate transporter is present, as in *E. acidaminophilum *[64]. Electrons from the oxidation of formate to CO_2 _are transferred directly to the hydrogenase *via *ferredoxin to reduce H^+ ^ions to hydrogen inside the cell, thus lowering the proton concentration. Genes encoding the hydrogenase maturation proteins HydE, HydF, and HydG are localized downstream from the hydrogenase gene *hydA*.

### The glycine synthase/glycine reductase pathway and the Wood-Ljungdahl pathway: a surprising combination

A particular metabolic feature of *C. sticklandii *is the simultaneous presence of genes encoding the complete Wood-Ljungdahl pathway and the glycine synthase (reversed gcv system)/glycine reductase pathway (Figure 1A). The Wood-Ljungdahl pathway is the main characteristic feature of acetogenic bacteria, making them capable of using CO_2 _as an electron acceptor. Many of these organisms can also grow autotrophically utilizing CO_2_. In certain purine- or amino acid-fermenting "acetogenic" bacteria acetate is synthesized from C1-carbon units *via *the glycine synthase/glycine reductase pathway [65,66]. It has been reported that the glycine synthase/glycine reductase pathway is not present in acetogenic bacteria containing the Wood-Ljungdahl pathway [67]. Since *C. sticklandii *possesses both pathways, a possible function of the Wood-Ljungdahl pathway may be the formation of methylene-THF from CO_2_, which is then used in the glycine synthase/glycine reductase pathway as described for amino acid-fermenting "acetogenic" bacteria. However, this does not lead to a net energy conservation, since ATP formed *via *the glycine reductase reaction is consumed for the activation of formate to form formyl-THF. It also seems likely that the whole Wood-Ljungdahl pathway is turned off completely under fermentative conditions [68], since metabolites involved in fermentation can act as terminal electron acceptors, thus removing the electrons necessary for the reduction of CO_2 _to acetate. Therefore, we assume that *C. sticklandii *cannot grow autotrophically like acetogenic bacteria.

Two main processes of chemiosmotic energy conservation have been described in acetogenic bacteria. The first, observed in *Moorella thermoacetica*, assumes that the methylene-THF reductase is associated with the cytoplasmic membrane and is coupled to a proton translocating NADH:quinone oxidoreductase with cytochromes and menaquinone. The second energy conservation system, described in *A. woodii*, suggests that the methyl group transfer from methyl-THF to the carbon monoxide dehydrogenase/acetyl-CoA synthase by a membrane-bound corrinoid protein is accompanied by an efflux of Na^+ ^ions. In both systems energy is conserved by the ion-motive force-driven ATPase system. In *C. sticklandii *the methylene-THF reductase does not seem to be attached to the cytoplasmic membrane, cytochromes are missing, menaquinone is not synthesized and membrane-bound corrinoids are absent. Recently, Schmidt *et al. *proposed that in *A. woodii *energy could be conserved in the Wood-Ljungdahl pathway by the membrane-bound Rnf reaction [59]. As mentioned above, the Rnf complex catalyzes an electron flow from reduced ferredoxin to NAD^+^, a reaction coupled to proton or sodium efflux. Reduced NAD^+ ^often provides electrons for the reductive steps of the methyl-branch of the Wood-Ljungdahl pathway. The regeneration of NADH *via *the reduced ferredoxin-dependent Rnf complex leads to chemiosmotic energy conservation. Appropriate experiments are necessary to determine whether either the Wood-Ljungdahl pathway or the glycine synthase/glycine reductase pathway, or both, are active in *C. sticklandii*.

In any case, it was interesting to see whether other microorganisms contain this particular combination. For this purpose, the characteristic protein sequences of the carbon monoxide dehydrogenase/acetyl-CoA synthase from the Wood-Ljungdahl pathway, the gcv- system and the glycine reductase from *C. sticklandii *were compared with completely sequenced bacterial genomes and those available as a draft. This analysis reveals that, in addition to *C. sticklandii*, only four bacterial species contain the genes coding for these proteins (Additional file 8): *Alkaliphilus metalliredigens*, *Carboxydothermus hydrogenoformans*, *Clostridium carboxidivorans*, and several strains of *Clostridium difficile*. Of these *C. hydrogenoformans *can grow autotrophically using carbon monoxide as the sole carbon and energy source and presumably synthesizes acetate from CO_2 _*via *the Wood-Ljungdahl pathway [69,70]. Altogether, these results clearly demonstrate that the coexistence of genes encoding the Wood-Ljungdahl pathway and the glycine synthase/glycine reductase pathway, as found in *C. sticklandii*, is not widespread.

## Conclusions

Sequencing and annotation of the small genome of *C. sticklandii *confirms that this organism derives most of its carbon and energy from the fermentation of amino acids. In particular, the genes coding for D-proline and glycine reductases, characteristic enzymes of the Stickland reaction, are both present in this genome. The inspection of the D-proline reductase cluster leads to the discovery that PrdC is a selenoprotein and contributes to a detailed understanding of the mechanism of D-proline reductase. Analysis of amino acid utilization demonstrates that *C. sticklandii *degrades amino acids in a preferential way. The data generated shows that threonine, arginine and serine are the best sources of carbon and energy, whereas aromatic amino acids, glutamate, aspartate and alanine are not or hardly utilized. The degradation of lysine, a process which has been described as a major energy source, is only performed in the stationary growth phase. The genome-scale metabolic reconstruction of *C. sticklandii *provides valuable information about energy conservation in anaerobic microorganisms. Beside SLP, the characteristic energy-conserving method in fermentative bacteria, *C. sticklandii *possesses several systems to conserve energy by ETP. These include a membrane-associated ion pumping Rnf complex as well as the rather unusual combination of a F- and V-type ATPase. Another particular feature is the simultaneous presence of two CO_2_-fixation pathways: the Wood-Ljungdahl and the glycine synthase/glycine reductase pathways. Comparative genomics reveals that the amino acid degradation pathways of *C. sticklandii *present homologies with the environmental bacteria, *A. metalliredigens *and *A. oremlandii *OhILAs, and with the pathogenic clostridial strains, *C. botulinum *and especially *C. difficile*. Since *C. diffcile *shares a similar energy metabolism with *C. sticklandii*, these two organisms could serve as an excellent model for comparative studies of the lifestyle of pathogenic and non-pathogenic organisms.

## Methods

### Genome sequencing and assembly

A Sanger/pyrosequencing hybrid approach was used for whole-genome sequencing of *C. sticklandii *DSM 519. A shotgun library was constructed with a 10 kb size fraction obtained by mechanical shearing of total genomic DNA. The fragments were cloned into vector pCNS (pSU18 derived). Insert ends of the recombinant plasmids (21504 reads, ~6× coverage) were sequenced by dye terminator chemistry using ABI3730 sequencers. Next, total genomic sheared DNA was sequenced (~21× coverage) using the Roche GS20 sequencer. For the assembly, we used the Arachne "HybridAssemble" (Broad Institute, http://www.broad.mit.edu) that combines the 454 contigs with Sanger reads.

### Gene prediction and annotation

Annotation was performed using MaGe [44,71], which allows graphic visualization of the *C. sticklandii DSM 519 *annotations enhanced by a simultaneous representation of synteny groups in other genomes chosen for comparisons. Coding sequences (CDS) likely to encode proteins were predicted with AMIGene [72].

### Growth conditions and amino acid analysis

*C. sticklandii *strain DSM 519 was grown at 37°C under a nitrogen atmosphere in the synthetic medium described by Wagner and Andreesen which contains required trace elements such as selenite and tungstate [33]. Cultures for the analysis of amino acid utilization were prepared in a medium containing all amino acids (except alanine and aspartate) at a concentration of 2 mM, as well as the cofactor and trace element solutions used in the synthetic medium [33]. Samples were collected at different growth phases (exponential, the end of the exponential, stationary, and the end of the stationary phases), centrifuged for 10 min at 20,000 *g*, and the supernatant was analyzed by LC-MS. The majority of the amino acids were analyzed directly and some were derivatized with o-phthalic aldehyde and N-acetyl L-cysteine as described by Brückner *et al. *[73]. Additional file 5 shows the amino acid utilization profiles obtained.

### Mass spectrometry

Chromatographic separations of free amino acids were performed using an Aquity UPLC BEH C18 column (150 × 2.1 mm, 1.7 μm; Waters). The amino acids were eluted with 10 mM ammonium acetate solution containing 0.1% formic acid (A) and methanol containing 0.1% formic acid (B). Chromatographic separations of the derived amino acids were performed employing an XBridge C18 column (150 × 2.1 mm, 5 μm; Waters). These were eluted with a 30 mM ammonium acetate solution (A) and methanol (B). All LC-MS analyses were performed using a LTQ/Orbitrap high resolution mass spectrometer coupled to an Accella LC system (Thermo-Fisher Scientific).

### Nucleotide sequence accession number

The genome sequence reported in this article has been deposited in the EMBL database [EMBL:FP565809]. The genome annotation and features are available at http://www.genoscope.cns.fr/agc/microscope/Anaeroscope.

## List of abbreviations

ATP: adenosine triphosphate; CoA or CoASH: coenzyme A; ETP: electron-transport phosphorylation; fd: ferredoxin; FMN: flavin mononucleotide; gcv-system: glycine cleavage system; LC-MS: Liquid Chromatography-Mass Spectrometry; MaGe: Magnifying Genomes; NADH: reduced nicotinamide adenine dinucleotide; NADPH: reduced nicotinamide adenine dinucleotide phosphate; Ppase: pyrophosphatase; Rnf: protein involved in *Rhodobacter capsulatus *nitrogen fixation; Sec: selenocysteine; SLP: substrate-level phosphorylation; THF: tetrahydrofolate; WGS: whole genome shotgun.

## Authors' contributions

AK, NF and GNC performed genome and comparative analysis and coordinated experimental procedures. SC carried out the growth experiments. ST analysed amino acid utilization by LC-MS. NP contributed to the experimental procedures. AL contributed to elaboration of the clostridial database Anaeroscope. MG constructed shotgun library for sequencing. VB carried out the assembly and finishing of the genome. EP performed bioinformatic analyses. ST, AL and VB participated in writing sections of the manuscript. MS, DL, and JW critically reviewed the manuscript. GNC and JRA contributed to writing of the manuscript. AK and NF wrote the manuscript. All authors read and approved the final manuscript.

## Supplementary Material

Additional file 1 **Genomic and metabolic features of *C. sticklandii *compared with other clostridia**. *All data are extracted from the MaGe annotations except those of *Clostridium sporogenes*, which are from the NCBI database*. *C.stick: *Clostridium sticklandii *DSM 519; C.acet: *Clostridium acetobutylicum *ATCC 824; C.beij: *Clostridium beijerinckii *NCIMB 8052; C.botu*: Clostridium botulinum *A Hall; C.diff: *Clostridium difficile *630; C.kluv: *Clostridium kluyveri *DSM 555; C.novy: *Clostridium novyi *NT; C.perf: *Clostridium perfringens *ATCC 13124; C.phyt: *Clostridium phytofermentans *ISDg; C.teta: *Clostridium tetani *E88; C.ther: *C. thermocellum *ATCC 27405; C.spor: *C. sporogenes *ATCC 1557; A.meta: *Alkaliphilus metalliredigens *QYMF; A.orem: *Alkaliphilus oremlandii *OhILAs; M.ther: *Moorella thermoacetica *ATCC 39073*. *2-ketoacid ferredoxin oxidoreductases. Click here for file

Additional file 2 ***C. sticklandii *synteny conservation and Bidirectional Best Hit percentages with the most closely related clostrial species**. Synteny conservation is given by the percentage of CDS in synteny between *C. sticklandii *and the complete sequenced genomes. Click here for file

Additional file 3 **Characteristic gene products of *C. sticklandii*, their protein symbols and their corresponding labels**. This is a listing of all genes/proteins that are discussed in the text. Click here for file

Additional file 4 **Comparative analysis of amino acid degradation pathways in several clostridial species**. *Blast searches (criteria: 30% identity over at least 80% of the length of the reference protein) were performed to determine the presence of the key enzymes in the microorganisms. All protein sequences were taken from *C. sticklandii *with two exceptions: the sequence of methylaspartate mutase was from *Clostridium cochlearium*, and that of 2-hydroxyglutaryl-CoA dehydratase from *Acidaminococcus fermentans*. C.stick: *Clostridium sticklandii *DSM 519; C.acet: *Clostridium acetobutylicum *ATCC 824; C.beij: *Clostridium beijerinckii *NCIMB 8052; C.botu*: Clostridium botulinum *A Hall; C.diff: *Clostridium difficile *630; C.kluv: *Clostridium kluyveri *DSM 555; C.novy: *Clostridium novyi *NT; C.perf: *Clostridium perfringens *ATCC 13124; C.phyt: *Clostridium phytofermentans *ISDg; C.teta: *Clostridium tetani *E88; C.ther: *C. thermocellum *ATCC 27405; C.spor: *C. sporogenes *ATCC 15579; A.meta: *Alkaliphilus metalliredigens *QYMF; A.orem: *Alkaliphilus oremlandii *OhILAs; M.ther: *Moorella thermoacetica *ATCC 39073; C.coch: *Clostridium cochlearium*; A.ferm: *Acidaminococcus fermentans *DSM 20731. The genome of *Clostridium cochlearium *is not yet sequenced*. Click here for file

Additional file 5 **LC-MS analyses of amino acid utilization during growth**. For each amino acid, its presence in the medium was checked at different growth phases (colour graphs). The growth kinetic is represented by a yellow graph. Citrulline, ornithine, alanine and aspartate (indicated by a black frame) were not added to the medium. They appeared as intermediates or products of metabolism from other amino acids. Since the utilization of methionine was not discussed in the article, the graph for this amino acid is not shown. Click here for file

Additional file 6 **Cell growth of *C. sticklandii *in three types of media with different amino acid composition**. Graph 1: medium containing amino acids that are catabolized in the exponential phase (Pro, Asn, Thr, Ser, Arg, Cys); Graph 2: medium containing amino acids that are catabolized in the exponential and stationary phase (Pro, Asn, Thr, Ser, Arg, Cys, Leu, Iso, Met, Gln, His, Lys); Graph 3: the amino acid combination of this medium is equivalent to the second medium, complemented with amino acids apparently not metabolized (Pro, Asn, Thr, Ser, Arg, Cys, Leu, Iso, Met, Gln, His, Lys, Trp, Val, Phe, Glu). Tyrosine was added to each medium. Click here for file

Additional file 7 **Comparison of the D-proline reductase gene cluster from different clostridial species**. *White arrows indicate hypothetical proteins or those presumed not to be involved in the D-proline reductase reaction. The letter "U" indicates the presence of selenocysteine. In *Clostridium botulinum A Hall, *a part of the cluster is duplicated*. Click here for file

Additional file 8 **Presence of characteristic enzymes of the glycine synthase (Gcv proteins)/glycine reductase (Grd proteins) and Wood-Ljungdahl (CODH proteins) pathway in microbial genomes**. Complete and WGS microbial genomes (as of May 6th, 2010) were scanned for some selected characteristic orthologous genes coding for carbon monoxide dehydrogenase/acetyl-CoA synthetase (Wood-Ljungdahl pathway), glycine reductase (Stickland reaction), and the glycine synthase (gcv-system) using a blastx search with stringent criteria (40% of positive residues over at least 80% of the length of the reference protein). For each target genome, the presence/absence of each protein was summarized in a 1/0 vector. Those WGS microbial genomes which do not contain all genes of both pathways are omitted. Click here for file
