# Supplementary material for: Clostridium sticklandii, a specialist in amino acid degradation:revisiting its metabolism through its genome sequence
Source: BMC Genomics. 2010 Oct 11;11:555. doi: 10.1186/1471-2164-11-555 (PMC3091704; doi:10.1186/1471-2164-11-555)
Supplement: Additional file 1 — Genomic and metabolic features of C. sticklandii compared with other clostridia. All data are extracted from the MaGe annotations except those of Clostridium sporogenes, which are from the NCBI database. C.stick: Clostridium sticklandii DSM 519; C.acet: Clostridium acetobutylicum ATCC 824; C.beij: Clostridium beijerinckii NCIMB 8052; C.botu: Clostridium botulinum A Hall; C.diff: Clostridium difficile 630; C.kluv: Clostridium kluyveri DSM 555; C.novy: Clostridium novyi NT; C.perf: Clostridium perfringens ATCC 13124; C.phyt: Clostridium phytofermentans ISDg; C.teta: Clostridium tetani E88; C.ther: C. thermocellum ATCC 27405; C.spor: C. sporogenes ATCC 1557; A.meta: Alkaliphilus metalliredigens QYMF; A.orem: Alkaliphilus oremlandii OhILAs; M.ther: Moorella thermoacetica ATCC 39073. *2-ketoacid ferredoxin oxidoreductases. [file 1471-2164-11-555-S1.DOC]

Genomic features of clostridia

|  | **C.stick** | C.acet | C.beij | C.botu | C.diff | C.kluv | C.novy | C.perf | C.phyt | C.teta | C.ther | C.spor | A.meta | A.orem | M.ther |
| --- | --- | --- | --- | --- | --- | --- | --- | --- | --- | --- | --- | --- | --- | --- | --- |
| Chromosome size (Mb) | **2.7** | 3.9 | 6 | 3.7 | 4.3 | 3.9 | 2.5 | 3 | 4.8 | 2.7 | 3.8 | 4.1 | 4.9 | 3.1 | 2.6 |
| Plasmid size (pb) | **-** | 192,000 | - | 16,344 | 7,881 | 59,182 | - | 54,310 | - | 74,082 | - | - | - | - | - |
| G+C content (%) | **33.3** | 30.9 | 29.8 | 28.1 | 29.1 | 32 | 28.8 | 28.5 | 35.3 | 28.7 | 39 | 28.1 | 36.8 | 36.2 | 55.7 |
| Repeat regions (%) | **2.13** | 4.9 | 8.3 | 5.7 | 9.8 | 8.4 | 5.8 | 3.4 | 3.8 | 4.8 | 10.3 | 6.2 | 10.2 | 3.6 | 3.1 |
| Number of CDS | **2573** | 3906 | 5597 | 3584 | 3920 | 4290 | 2396 | 2738 | 4648 | 2824 | 3888 | 3767 | 5531 | 3227 | 2712 |
| Protein coding density (%) | **89.2** | 86 | 79.8 | 80.7 | 82 | 84.7 | 86.8 | 82.8 | 82.1 | 88.4 | 84.2 | 82 | 83.0 | 84.4 | 87.5 |
| Average CDS length (bp) | **945** | 888 | 872 | 855 | 909 | 798 | 928 | 925 | 879 | 885 | 861 | 898 | 776 | 837 | 862 |
| Number of tRNAs | **59** | 72 | 94 | 81 | 87 | 61 | 81 | 96 | 60 | 54 | 56 | 77 | 104 | 86 | 51 |
| Number of rRNA clusters | **6** | 11 | 14 | 8 | 11 | 7 | 10 | 10 | 8 | 6 | 4 | 7 | 10 | 8 | 1 |

Metabolic features of clostridia

|  | **C.stick** | C.acet | C.beij | C.botu | C.diff | C.kluv | C.novy | C.perf | C.phyt | C.teta | C.ther | C.spor | A.meta | A.orem | M.ther |
| --- | --- | --- | --- | --- | --- | --- | --- | --- | --- | --- | --- | --- | --- | --- | --- |
| Sulfate assimilation | **-** | + | + | - | - | + | - | - | + | - | + | - | + | - | - |
| Glycine reductase | **+** | - | - | + | + | - | - | - | - | - | - | + | + | + | - |
| Proline reductase | **+** | - | - | + | + | - | - | - | - | - | - | + | + | + | - |
| Number of ferredoxin oxidoreductases^*^/ Mb | **2.6** | 1 | 0.6 | 0.8 | 1.2 | 0.7 | 0.8 | 0.3 | 0.8 | 1.1 | 1.5 | 0.7 | 1.8 | 2.6 | 2.6 |
| Rnf complex | **+** | **-** | **+** | **+** | **+** | **+** | **+** | **+** | **+** | **+** | **+** | **+** | **+** | **+** | **-** |
