# Supplementary material for: Clostridium sticklandii, a specialist in amino acid degradation:revisiting its metabolism through its genome sequence
Source: BMC Genomics. 2010 Oct 11;11:555. doi: 10.1186/1471-2164-11-555 (PMC3091704; doi:10.1186/1471-2164-11-555)
Supplement: Additional file 2 — C. sticklandii synteny conservation and Bidirectional Best Hit percentages with the most closely related clostrial species. Synteny conservation is given by the percentage of CDS in synteny between C. sticklandii and the complete sequenced genomes. [file 1471-2164-11-555-S2.DOC]

| **Strain** | **CDS in synteny (%)** | **Bidirectional Best Hits (%)** |
| --- | --- | --- |
| *A. metalliredigens* QYMF | 46.9 | 65.1 |
| *A. oremlandii OhILAs* | 39.4 | 56.9 |
| *C. difficile 630* | 38.8 | 56.8 |
| *C. botulinum* A Hall | 36.8 | 56.4 |
| *C. sporogenes* ATCC 15579 | 36.8 | 56.9 |
| *C. beijerinckii* NCIMB 8052 | 35.0 | 57.5 |
| *Thermoanaerobacter* sp. X514 | 33.9 | 46.9 |
| *T. tengcongensis* MB4 | 32.9 | 46.6 |
| *C. novyi* NT | 32.2 | 47.6 |
| *C. kluyveri* DSM 555 | 32.1 | 52.3 |
