# Supplementary material for: Clostridium sticklandii, a specialist in amino acid degradation:revisiting its metabolism through its genome sequence
Source: BMC Genomics. 2010 Oct 11;11:555. doi: 10.1186/1471-2164-11-555 (PMC3091704; doi:10.1186/1471-2164-11-555)
Supplement: Additional file 3 — Characteristic gene products of C. sticklandii, their protein symbols and their corresponding labels. This is a listing of all genes/proteins that are discussed in the text. [file 1471-2164-11-555-S3.DOC]

| **Characteristic gene products** | **Protein symbol** | **Label** |
| --- | --- | --- |
| **Sec insertion machinery**: |  |  |
| Selenocysteine synthase | SelA | CLOST_1358 |
| Selenocystein-specific elongation factor | SelB | CLOST_1357 |
| Selenophosphate synthase | SelD | CLOST_1359 |
| SeC(p) tRNA | SelC | CLOST_tRNA59 |
| **Proline reductase:** |  |  |
| D-Proline reductase proprotein | PrdA | CLOST_2234 |
| D-Proline selenoprotein | PrdB | CLOST_2232 |
| Sec-containing electron transfer protein | PrdC | CLOST_2236 |
| **Glycine reductase:** |  |  |
| Substrate-specific activating selenoprotein | GrdB | CLOST_1113 CLOST_1114 |
| GrdB-stabilizing proprotein | GrdE | CLOST_1110 |
| Redox-active selenoprotein forming a carboxymethyl-selenoether | GrdA | CLOST_1112  CLOST_1111 |
| Protein forming a protein-bound acetyl-ester from the GrdA-carboxymethyl-selenoether | GrdC | CLOST_1115 |
| Protein releasing the protein-bound acetyl group as acetyl-phosphate | GrdD | CLOST_1116 |
| **Glycine cleavage system:** |  |  |
| Aminomethyltransferase | GcvT | CLOST_0426 |
| Lipoylprotein | GcvH | CLOST_0427  CLOST_1127 |
| Glycine dehydrogenase | GcvP | CLOST_0428  CLOST_0429 |
| Lipoamide dehydrogenase | GcvL | CLOST_1166 |
| **Threonine catabolism pathways:** |  |  |
| Threonine dehydrogenase | Tdh | CLOST_1621 |
| Threonine aldolase | ItaE | CLOST_0572 |
| Threonine dehydratase | TdcB | CLOS__0395 |
| **Arginine deiminase pathway:** |  |  |
| Arginine deiminase | ArcA | CLOST_0926 |
| Ornithine carbamoyltransferase | ArcB | CLOST_0927 |
| Carbamate kinase | ArcC | CLOST_0928 |
| **Ornithine reductive pathway:** |  |  |
| Ornithine cyclodeaminase | ArcB | CLOST_1603 |
| Proline racemase | PrdF | CLOST_2228 |
| **Ornithine oxidative pathway:** |  |  |
| Ornithine racemase | Orr | CLOST_1288 |
| Ornithine aminomutase | OraE  OraS | CLOST_1290  CLOST_1291 |
| 2,4-diaminopentanoate dehydrogenase | Ord | CLOST_1294 |
| 2-amino-4-ketopentanoate thiolase (α and β subunits) | OrtA  OrtB | CLOST_1292  CLOST_1293 |
| **Lysine fermentation pathway:** |  |  |
| L-lysine 2,3-aminomutase | KamA | CLOST_1382 |
| β-L-lysine-5,6-aminomutase (α and β subunits) | KamD  KamE | CLOST_1379  CLOST_1378 |
| 3,5−diaminohexanoate dehydrogenase | Kdd | CLOST_1383 |
| 3−keto-5-aminohexanoate cleavage enzyme | Kce | CLOST_1384 |
| 3-aminobutyryl-CoA ammonia lyase | Kal | CLOST_1385 |
| Acetoacetate:butyrate CoA transferase (α and β subunits) | AtoA  AtoD | CLOST_1124  CLOST_1123 |
| **Serine catabolism pathway:** |  |  |
| L-serine dehydratase | SdhA  SdhB | CLOST_1364  CLOST_1365 |
| **Cysteine catabolism pathway:** |  |  |
| Putative L-cysteine sulphide lyase |  | CLOST_2039 |
| **Butyrate fermentation (from acetyl-CoA):** |  |  |
| Acetyl-CoA acetyl transferase | AtoB | CLOST_1134 |
| 3-Hydroxybutyryl-CoA dehydrogenase | Hbd | CLOST_1133 |
| Crotonase | Crt | CLOST_1132 |
| Butyryl-CoA dehydrogenase | Bcd | CLOST_1135 |
| Electron transfer flavoprotein | EtfA  EtfB | CLOST_1137  CLOST_1136 |
| **Oxidative stress reponse:** |  |  |
| Mn-superoxide dismutase | SodA | CLOST_1948 |
| Superoxide reductase | SorA | CLOST_1779 |
| Alkyl hydroperoxide reductase | YkuU | CLOST_2030 |
| Glutathione peroxidase | BtuE | CLOST_0978  CLOST_2446 |
| Selenoperoxiredoxin | PrxU | CLOST_2406 |
| Thioredoxin dependent peroxidase |  | CLOST_1360 |
| Methionine sulfoxide reductase A | MsrA | CLOST_1083 |
| Methionine sulfoxide reductase B | MsrB | CLOST_2458 |
| Peroxide-responsive repressor | PerR | CLOST_1148 |
| **Wood-Ljungdahl pathway:** |  |  |
| Carbon monoxide dehydrogenase | CODH-beta | CLOST_1160 |
| Acetyl-CoA synthase | CODH-alpha | CLOST_1171 |
| **Rnf complex:** |  |  |
| Electron transport complex | RnfB | CLOST_1397 |
| Putative inner membrane subunit | RnfA | CLOST_1398 |
| Putative inner membrane NADH-quinone reductase | RnfE | CLOST_1399 |
| Electron transport complex protein precursor | RnfG | CLOST_1400 |
| Putative inner membrane oxidoreductase | RnfD | CLOST_1401 |
| Electron transport complex protein | RnfC | CLOST_1402 |
| **Hydrogenases and maturation proteins:** |  |  |
| Putative catalytic subunit of iron-only hydrogenase | HymC | CLOST_0907 |
| Putative iron-only hydrogenase, electron-transfer subunit | HymB | CLOST_0908 |
| Putative iron-only hydrogenase, electron-transfer subunit | HymA | CLOST_0909  CLOST_1663 |
| Periplasmic iron-only hydrogenase | HydA | CLOST_0839 |
| Iron-only hydrogenase maturation protein | HydF | CLOST_0845 |
| Fe-hydrogenase assembly protein | HydG | CLOST_0846 |
| Iron-only hydrogenase maturation protein | HydE | CLOST_0847 |
