# Supplementary material for: Clostridium sticklandii, a specialist in amino acid degradation:revisiting its metabolism through its genome sequence
Source: BMC Genomics. 2010 Oct 11;11:555. doi: 10.1186/1471-2164-11-555 (PMC3091704; doi:10.1186/1471-2164-11-555)
Supplement: Additional file 4 — Comparative analysis of amino acid degradation pathways in several clostridial species. Blast searches (criteria: 30% identity over at least 80% of the length of the reference protein) were performed to determine the presence of the key enzymes in the microorganisms. All protein sequences were taken from C. sticklandii with two exceptions: the sequence of methylaspartate mutase was from Clostridium cochlearium, and that of 2-hydroxyglutaryl-CoA dehydratase from Acidaminococcus fermentans. C.stick: Clostridium sticklandii DSM 519; C.acet: Clostridium acetobutylicum ATCC 824; C.beij: Clostridium beijerinckii NCIMB 8052; C.botu: Clostridium botulinum A Hall; C.diff: Clostridium difficile 630; C.kluv: Clostridium kluyveri DSM 555; C.novy: Clostridium novyi NT; C.perf: Clostridium perfringens ATCC 13124; C.phyt: Clostridium phytofermentans ISDg; C.teta: Clostridium tetani E88; C.ther: C. thermocellum ATCC 27405; C.spor: C. sporogenes ATCC 15579; A.meta: Alkaliphilus metalliredigens QYMF; A.orem: Alkaliphilus oremlandii OhILAs; M.ther: Moorella thermoacetica ATCC 39073; C.coch: Clostridium cochlearium; A.ferm: Acidaminococcus fermentans DSM 20731. The genome of Clostridium cochlearium is not yet sequenced. [file 1471-2164-11-555-S4.DOC]

| **Amino acid**  Enzyme | **C.stick** | **C.acet** | **C.beij** | **C.botu** | **C.diff** | **C.kluv** | **C.novy** | **C.perf** | **C.phyt** | **C.teta** | **C.ther** | **C.spor** | **A.meta** | **A.orem** | **A.ferm** |
| --- | --- | --- | --- | --- | --- | --- | --- | --- | --- | --- | --- | --- | --- | --- | --- |
| **Threonine** : |  |  |  |  |  |  |  |  |  |  |  |  |  |  |  |
| Threonine dehydrogenase | **+** |  |  |  | **+** |  |  |  |  |  |  |  | **+** | **+** |  |
| Threonine dehydratase | **+** |  | **+** | **+** | **+** | **+** | **+** | **+** | **+** | **+** |  | **+** | **+** | **+** | **+** |
| Threonine aldolase | **+** |  |  |  | **+** | **+** |  |  |  | **+** |  |  | **+** | **+** |  |
| **Serine** : |  |  |  |  |  |  |  |  |  |  |  |  |  |  |  |
| Serine dehydratase | **+** | **+** | **+** | **+** | **+** | **+** | **+** | **+** | **+** | **+** |  | **+** | **+** | **+** | **+** |
| **Arginine** : |  |  |  |  |  |  |  |  |  |  |  |  |  |  |  |
| Arginine deiminase | **+** |  |  | **+** |  |  |  | **+** |  |  |  | **+** | **+** |  |  |
| Ornithine carbamoyl-phosphate transferase | **+** | **+** | **+** | **+** | **+** | **+** | **+** | **+** | **+** |  | **+** | **+** | **+** |  | **+** |
| **Ornithine** : |  |  |  |  |  |  |  |  |  |  |  |  |  |  |  |
| 2,4-Diaminopentanoate  dehydrogenase | **+** |  |  |  | **+** |  |  |  |  |  |  |  | **+** | **+** |  |
| Ornithine cyclase | **+** |  |  | **+** | **+** |  |  |  |  |  |  | **+** |  |  |  |
| **Proline** : |  |  |  |  |  |  |  |  |  |  |  |  |  |  |  |
| Proline reductase  proprotein (PrdA) | **+** |  |  | **+** | **+** |  |  |  |  |  |  | **+** | **+** | **+** |  |
| **Lysine** : |  |  |  |  |  |  |  |  |  |  |  |  |  |  |  |
| 3,5-Diaminohexanoate  dehydrogenase | **+** |  |  |  |  |  |  |  |  |  |  |  | **+** | **+** |  |
| **Glycine** : |  |  |  |  |  |  |  |  |  |  |  |  |  |  |  |
| Glycine reductase (GrdA) | **+** |  |  | **+** | **+** |  |  |  |  |  |  | **+** | **+** | **+** |  |
| Glycine cleavage  enzyme (GcvPA) | **+** |  |  | **+** | **+** | **+** |  |  | **+** |  |  | **+** | **+** | **+** |  |
| **Histidine** : |  |  |  |  |  |  |  |  |  |  |  |  |  |  |  |
| Histidine ammonia lyase | **+** |  |  |  |  |  |  |  |  | **+** |  |  | **+** | **+** | **+** |
| **Glutamate** : |  |  |  |  |  |  |  |  |  |  |  |  |  |  |  |
| Methylaspartate mutase  (GlmS/GlmE) |  |  |  |  |  |  |  |  |  | **+** |  |  | **+** |  |  |
| 2-Hydroxyglutaryl-CoA  dehydratase (HgdA/HgdB) |  |  |  |  |  |  |  |  |  |  |  |  |  |  | **+** |
