# Supplementary figures and images for: Clostridium sticklandii, a specialist in amino acid degradation:revisiting its metabolism through its genome sequence
Source: BMC Genomics. 2010 Oct 11;11:555. doi: 10.1186/1471-2164-11-555 (PMC3091704; doi:10.1186/1471-2164-11-555)

## Slide 1
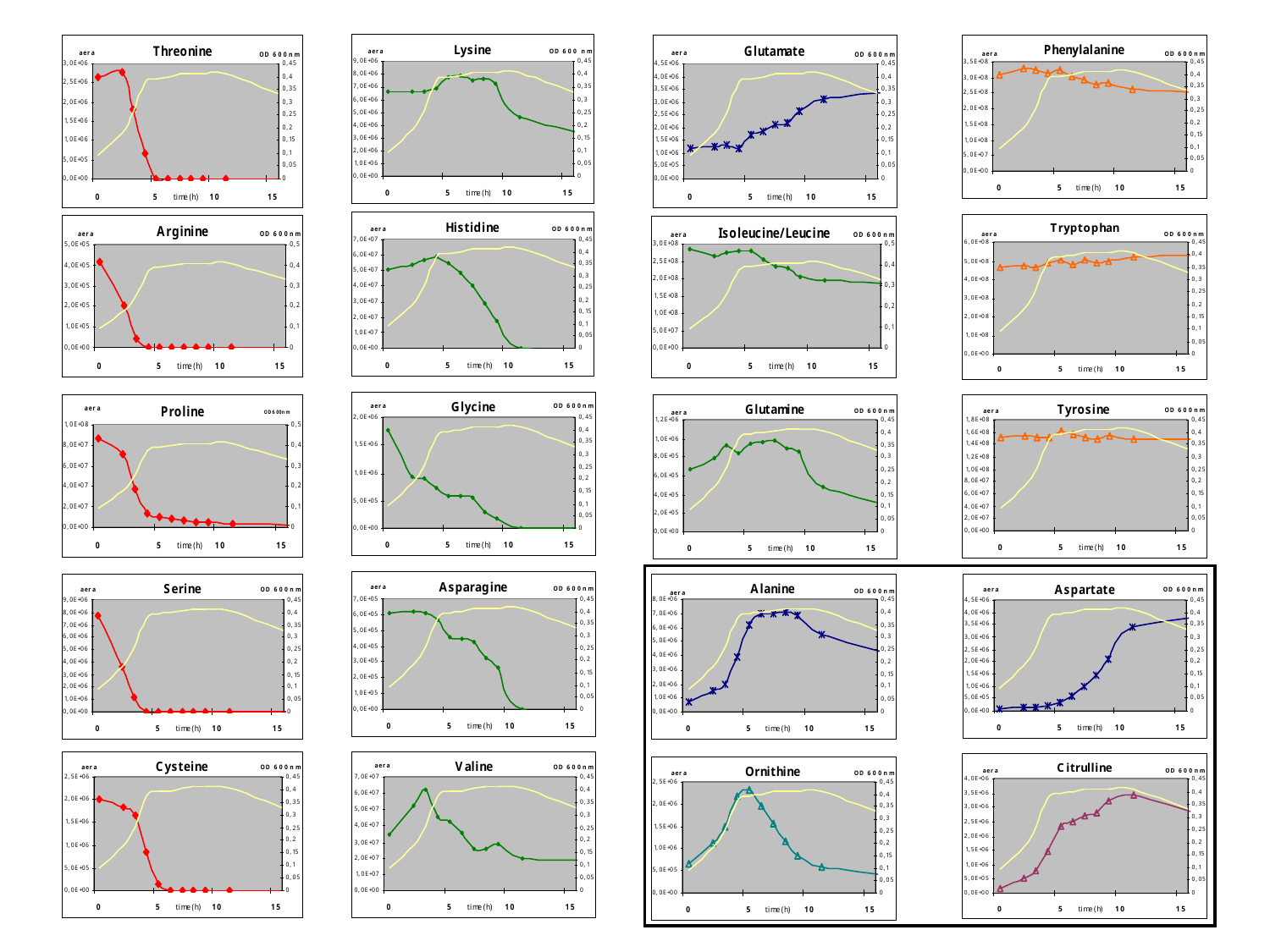

Supplement: Additional file 5 — LC-MS analyses of amino acid utilization during growth. For each amino acid, its presence in the medium was checked at different growth phases (colour graphs). The growth kinetic is represented by a yellow graph. Citrulline, ornithine, alanine and aspartate (indicated by a black frame) were not added to the medium. They appeared as intermediates or products of metabolism from other amino acids. Since the utilization of methionine was not discussed in the article, the graph for this amino acid is not shown. [file 1471-2164-11-555-S5.PPT]

## Slide 1
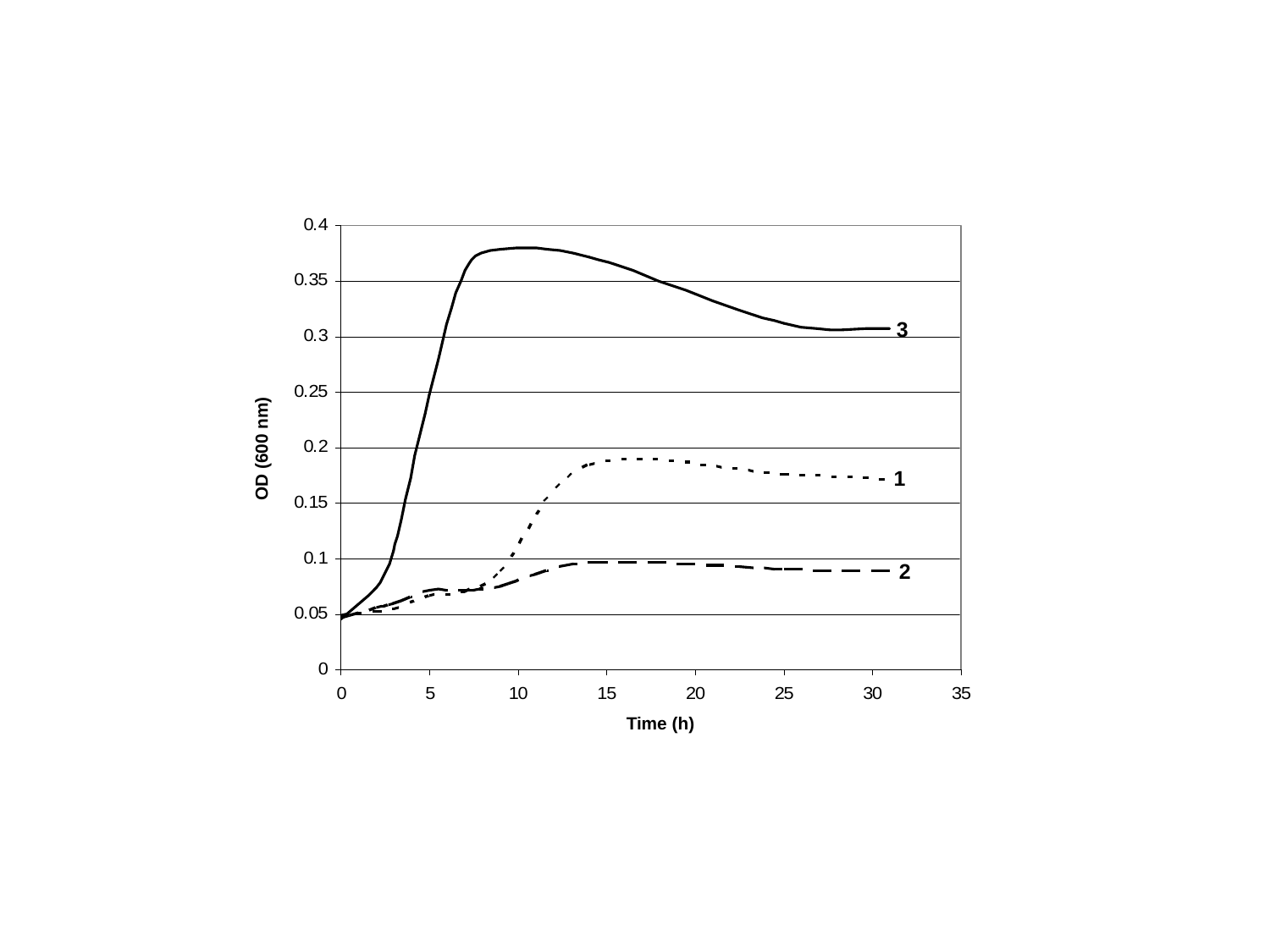

3
OD (600 nm)
1
2
Time (h)

Supplement: Additional file 6 — Cell growth of C. sticklandii in three types of media with different amino acid composition. Graph 1: medium containing amino acids that are catabolized in the exponential phase (Pro, Asn, Thr, Ser, Arg, Cys); Graph 2: medium containing amino acids that are catabolized in the exponential and stationary phase (Pro, Asn, Thr, Ser, Arg, Cys, Leu, Iso, Met, Gln, His, Lys); Graph 3: the amino acid combination of this medium is equivalent to the second medium, complemented with amino acids apparently not metabolized (Pro, Asn, Thr, Ser, Arg, Cys, Leu, Iso, Met, Gln, His, Lys, Trp, Val, Phe, Glu). Tyrosine was added to each medium. [file 1471-2164-11-555-S6.PPT]
