# Supplementary material for: Clostridium sticklandii, a specialist in amino acid degradation:revisiting its metabolism through its genome sequence
Source: BMC Genomics. 2010 Oct 11;11:555. doi: 10.1186/1471-2164-11-555 (PMC3091704; doi:10.1186/1471-2164-11-555)
Supplement: Additional file 7 — Comparison of the D-proline reductase gene cluster from different clostridial species. White arrows indicate hypothetical proteins or those presumed not to be involved in the D-proline reductase reaction. The letter "U" indicates the presence of selenocysteine. In Clostridium botulinum A Hall, a part of the cluster is duplicated. [file 1471-2164-11-555-S7.PPT]

## Slide 1
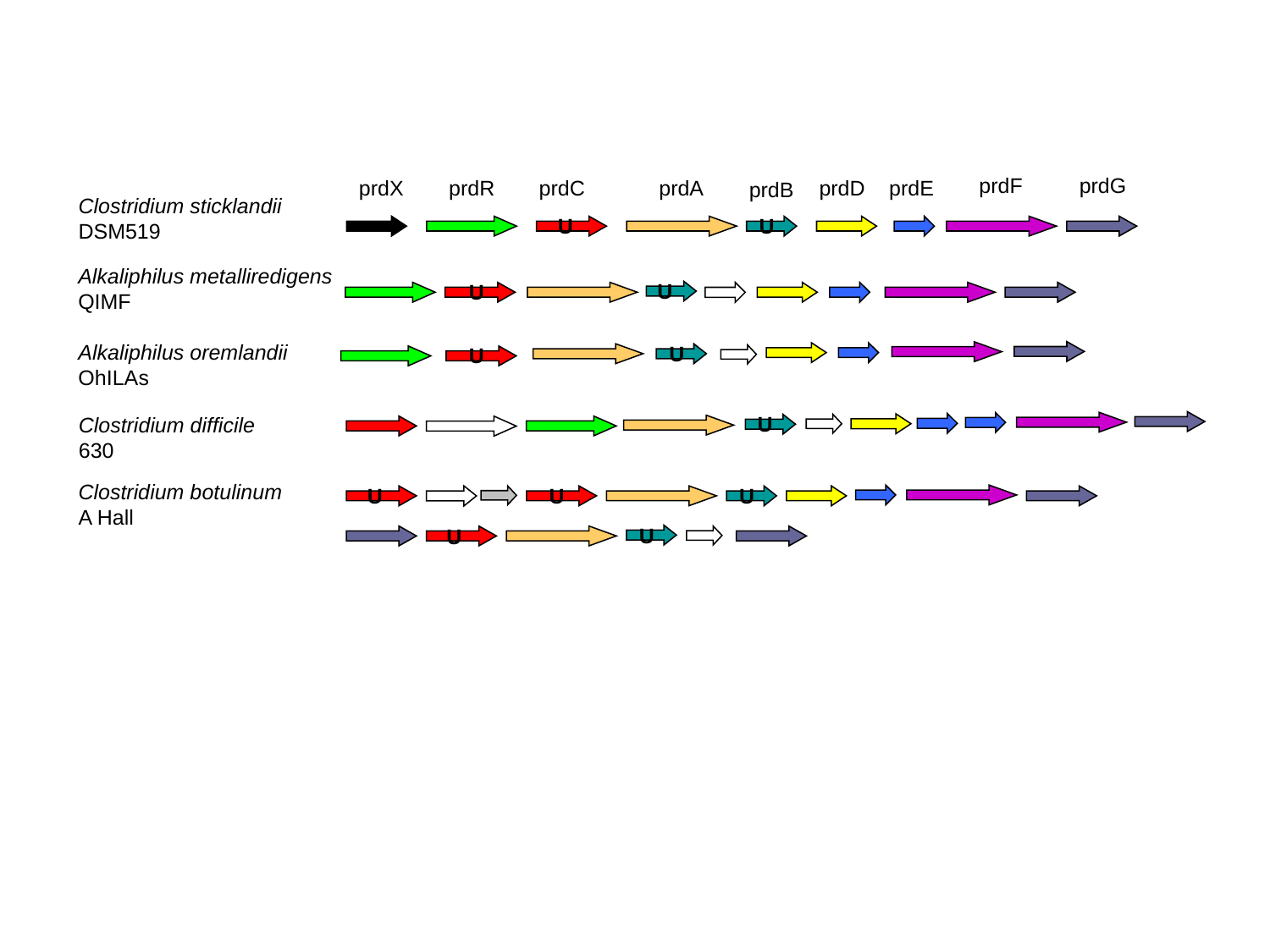

prdF
prdG
prdX
prdR
prdC
prdD
prdE
prdA
prdB
Clostridium sticklandii
DSM519
U
U
Alkaliphilus metalliredigens
QIMF
U
U
Alkaliphilus oremlandii
OhILAs
U
U
U
Clostridium difficile
630
Clostridium botulinum
A Hall
U
U
U
U
U
